# Chemical induction of hairpin RNAi molecules to silence vital genes in plant roots

**DOI:** 10.1038/srep37711

**Published:** 2016-11-29

**Authors:** Siming Liu, John I. Yoder

**Affiliations:** 1 Plant Sciences Department, University of California, Davis, CA 95616, USA.

## Abstract

Understanding the functions encoded by plant genes can be facilitated by reducing transcript levels by hairpin RNA (hpRNA) mediated silencing. A bottleneck to this technology occurs when a gene encodes a phenotype that is necessary for cell viability and silencing the gene inhibits transformation. Here we compared the use of two chemically inducible plant promoter systems to drive hpRNA mediated gene silencing in transgenic, hairy roots. We cloned the gene encoding the Yellow Fluorescence Protein (YFP) into the dexamethasone inducible vector pOpOff2 and into the estradiol induced vector pER8. We then cloned a hpRNA targeting YFP under the regulation of the inducible promoters, transformed *Medicago truncatula* roots, and quantified YFP fluorescence and mRNA levels. YFP fluorescence was normal in pOpOff2 transformed roots without dexamethasone but was reduced with dexamethasone treatment. Interestingly, dexamethasone removal did not reverse YFP inhibition. YFP expression in roots transformed with pER8 was low even in the absence of inducer. We used the dexamethasone system to silence acetyl-CoA carboxylase gene and observed prolific root growth when this construct was transformed into Medicago until dexamethasone was applied. Our study shows that dexamethasone inducibility can be useful to silence vital genes in transgenic roots.

Conditionally inducible vectors are useful for activating the expression of genes whose constitutive expression would have deleterious effects on the plant. For example the avrRPT2 gene, which in the presence of the RPS2 disease-resistance gene causes cell-death in *Arabidopsis thaliana*, could be transformed into an RPS2 plant by cloning the gene behind a chemically inducible promoter^1^. Transformation vectors with conditionally inducible elements also allow investigators to control the expression of transgenes to a particular stage during development, such as the controlled expression of a cell cycle gene during leaf maturation^2^. Transgene expression can be similarly restricted to particular plant parts by localized application of chemical inducers, therefore accomplishing spatially controlled expression^3^.

The most common chemically inducible expression systems are comprised of two components; a chimeric transcription factor that is activated by the inducer and a promoter that is recognized by the chimeric transcription factor^4^. A simple two component system is the ethanol responsive “alc system”, comprised of the AlcR transcription factor that is rapidly and reversibly induced by acetaldehyde and the AlcA promoter to which it binds^5^. Another type of two component systems useful in plants makes use of transcription factors fused to steroid receptors so that the transcription factor is only activated in the presence of the steroid^6^,^7^. In the dexamethasone system, it contains a primary transcription unit with the constitutive cauliflower mosaic virus (CaMV) 35S promoter that controls the expression of a chimeric transcription factor LhG4. LhG4 contains a high-affinity DNA-binding mutant of the lac repressor and the transcription activation domain of the Saccharomyces cerevisiae GAL^8^,^9^, which is fused to a rat glucocorticoid receptor making the resultant transcription factor, LhGR, activated by glucocorticoid steroids^10^. In its unliganded state, the LhGR protein is sequestered to the cytoplasm in a complex with the 90-kDa heat shock protein. In presence of dexamethasone inducer, LhGR protein binds to dexamethasone, leading to dissociation of HSP90 and the localization of the transcription factor to the nucleus where it binds the bidirectional promoter pOp6, the second transcription unit comprised of six lac operators that recruits RNA polymerase II to the cauliflower mosaic virus (CaMV) minimal 35S promoter (Pm35S)^10^,^11^.

The estrogen inducible system employs a chimeric transcription factor XVE comprised of the DNA binding domain of the bacterial repressor lexA, the transcription activating domain of VP16 and the human estrogen receptor ER^4^. The pER8 plant transformation vector contains a primary transcription unit with a strong constitutive promoter (pG10-90) that controls the coding region of XVE fusion protein. Similar to the dexamethasone system, the unliganded XVE protein remains cytoplasmic until bound to estradiol when it crosses into the nucleus. In presence of the inducer (β-estradiol), XVE protein binds to a second transcription unit containing eight copies of the LexA operators that recruits RNA polymerase II to the cauliflower mosaic virus (CaMV) minimal 35S promoter (Pm35S) to induce expression of the downstream targets, such as gene expression cassettes or RNA hairpin^4^.

Steroid inducible promoters have been used to drive the expression of short interfering (si) RNA molecules produced by dicer like cleavage of double stranded RNAs (dsRNA). The siRNAs direct Argonaute nucleases to cleave endogenous transcripts with sequence homology to the dsRNAs, leading to the silencing of the endogenous transcripts. The pHELLSGATE transformation vectors were developed to allow hpRNA constructs to be generated using the Gateway recombination system^12^. Combining the LhGR/pOp6 components with the Gateway compatible pHELLSGATE hpRNAi vectors led to the pOpOff family of inducible vectors^13^. The pER8 vector has also been modified to express hpRNAs upon chemical induction^14^.

*Agrobacterium rhizogenes* transformation is an effective tool for investigating the functions of genes encoding phenotypes in plant roots^15^. Genes of interest can be silenced and assayed in roots by using *A. rhizogenes* to introduce hpRNA constructs targeting candidate genes into roots^16^,^17^. In order to make transgenic roots expressing hpRNA constructions targeting genes necessary for root growth, we adapted existing steroid inducible transformation systems to chemically control hpRNA expression. In the current study we characterized two steroid inducible systems for their effectiveness at inducing gene silencing in hairy roots of Medicago. We cloned the fluorescent marker gene Yellow Fluorescence Protein (YFP) into both the dexamethasone inducible vector pOpOff2^13^ and the estradiol-inducible pER8^4^ systems. We then cloned a hpRNA targeting YFP behind the inducible promoter in each vector, allowing the chemical inducibility of RNAi to be monitored by YFP fluorescence. We introduced the modified vectors into Medicago roots by *Agrobacterium rhizogenes* mediated root transformation and monitored the kinetics and reversibility of YFP inhibition. We then showed that the dexamethasone system can be used to transform a hpRNA construction that targets a critical gene into Medicago roots and that chemical induction of this construct leads to a lethal phenotype. This study characterized the functionality of each modified system and tested their inducible silencing of a target gene, thereby providing a tool to investigate gene functions for root biology and fundamental RNAi process in plants.

## Results

### Dexamethasone and estradiol induce the attenuation of YFP fluorescence in a dose dependent manner

**Figure 1:**
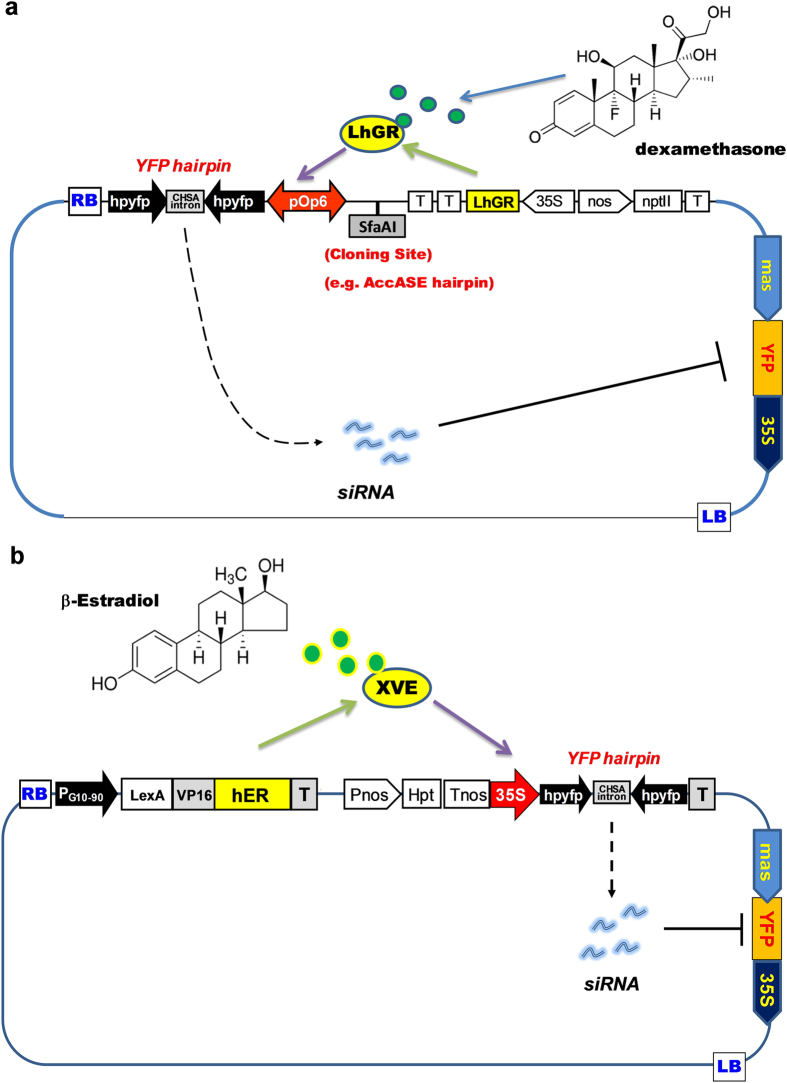
Diagram of pPopY-hpYFP and pER8Y-hpYFP chemically inducible vectors. (**a**) pOpOff2 vector was modified by adding a YFP gene driven by the mas promoter as well as a YFP hairpin cassette to the left side of the pOp6 promoter to make pPopY-hpYFP. This plasmid also contains a unique SfaAI site to the right of the promoter. T, terminator; LB, left border; RB, right border; nptII, kanamycin resistance gene; 35S, cauliflower mosaic virus minimal 35S promoter. (**b**) pER8 vector was modified by incorporating a YFP marker gene in the backbone and the YFP hairpin downstream of inducible promoter. This vector is called pER8Y in the absence of the hairpin and pER8Y-hpYFP in its presence. Hpt, hygromycin resistance gene; pG10-90, a constitutive active promoter.

We modified the pOpOff2 and pER8 vectors to contain a Yellow Fluorescence Protein (YFP) gene transcriptionally regulated by the mas promoter to use as a visual marker^18^. These vectors were named pPopY and pER8Y respectively. We then cloned a YFP hairpin cassette comprised of 373 nt inverted repeats of YFP coding sequence separated by an intron from the *Petunia hybrida* chalcone synthase A gene (CHS-A) intron behind the inducible promoter in each vector (Fig. 1a and b). These plasmids, named pPopY-hpYFP and pER8Y-hpYFP were then transformed into *M. truncatula* roots by *A. rhizogenes* transformation^15^. These constructs provide a means to visually monitor the effectiveness of chemical induction of RNAi in roots.

**Figure 2:**
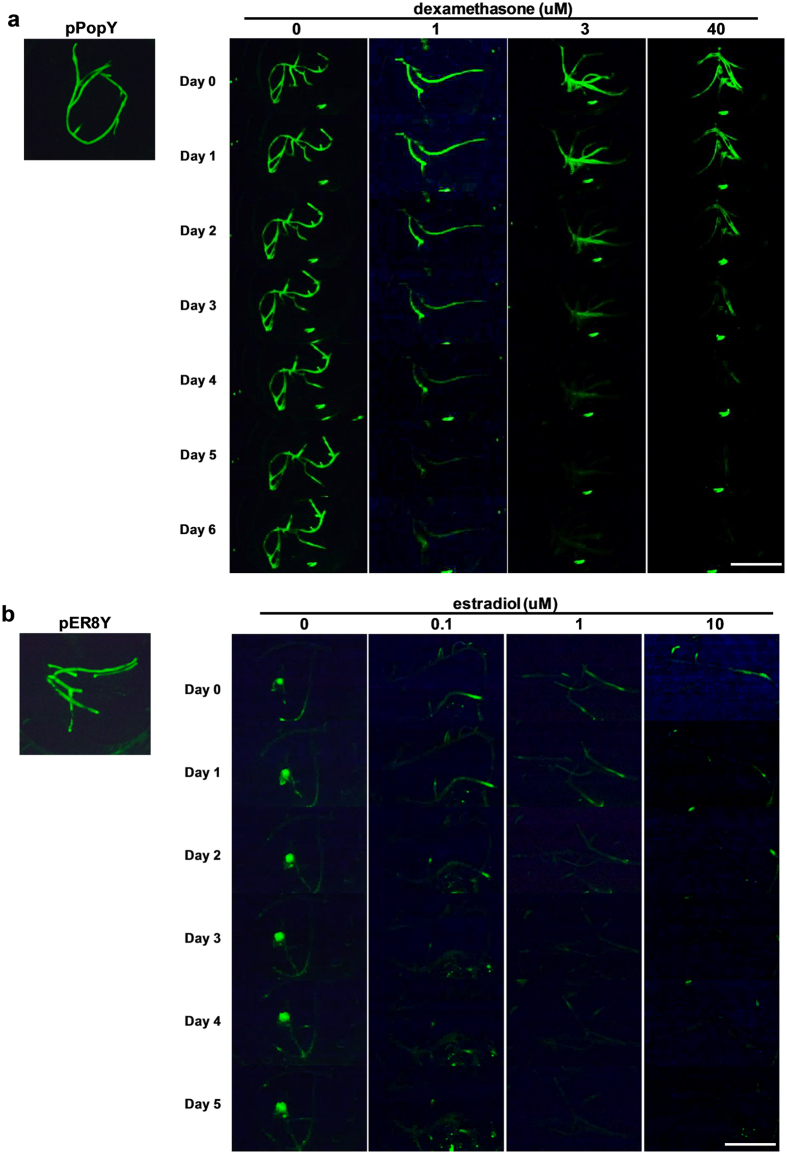
Treatment with dexamethasone and estradiol induced the attenuation of YFP fluorescence. (**a**) YFP fluorescence in roots transgenic for pPopY-hpYFP before and after exposure to 0, 1, 3, 40 uM Dex. Left inset: image of a root transformed with pPopY treated with Dex at day 5. Scale bar = 10 mm. (**b**) YFP fluorescence in pER8Y-hpYFP roots treated with 0, 0.1, 1, 10 uM estradiol. Left inset: image of the root transformed with pER8Y treated with estradiol at day 4. Scale bar = 10 mm.

Transgenic Medicago roots containing either pPopY, pPopY-hpYFP, pER8Y or pER8Y-hpYFP were treated with various concentrations of dexamethasone (Dex) or estradiol respectively, photographed YFP fluorescence daily, and fluorescence quantified using ImageJ. In the absence of Dex, the roots transformed with either pPopY or pPopY-hpYFP had similar YFP fluorescence levels (Fig. 2a), suggesting negligible expression of hpYFP in the absences of inducer. In contrast, pER8Y-hpYFP roots had reduced levels of YFP fluorescence compared to pER8Y roots even in the absence of the estradiol inducer (Fig. 2b).

**Figure 3:**
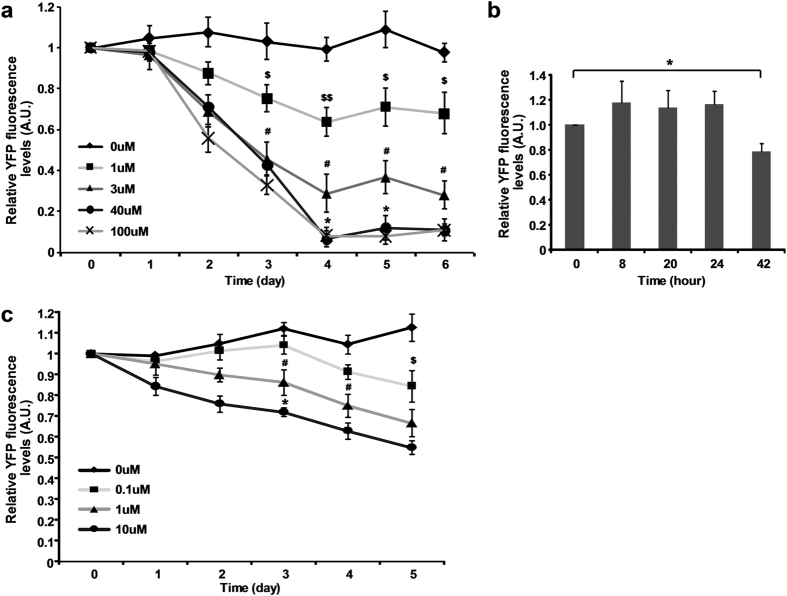
Quantification of YFP fluorescence after induction. (**a**) Quantitation of fluorescence after treatment with 0, 1, 3, 40, 100 uM Dex over 6 days. YFP fluorescence levels were quantified using Image J software, expressed relative to basal levels from day 0, averaged and plotted. Data expressed as mean +/− sem, n = 6. ^$^*p* < 0.05, ^$$^*p* < 0.01, 0 vs. 1 uM; ^#^*p* < 0.05, 1 vs. 3 uM; *p < 0.05, 3 vs. 40 uM at indicated time points, by t-test. (**b**) Quantitation of YFP fluorescence after treatment with 3 uM Dex over 42 hours. YFP fluorescence levels were quantified using Image J software, expressed relative to basal levels from hr 0, averaged and plotted.Data expressed as mean +/− sem, n = 6. *p < 0.05, 0 vs. 42 hr by t-test. (**c**) Quantitation of YFP fluorescence after treatments of 0, 0.1, 1, 10 uM estradiol. YFP fluorescence levels were quantified using Image J software, expressed relative to basal levels from day 0, averaged and plotted. Data expressed as mean +/− sem, n = 4. ^$^*p* < 0.05, 0 vs. 0.1 uM; ^#^*p* < 0.05, 0.1 vs. 1 uM; *p < 0.05, 1 vs. 10 uM at indicated time points, by t-test. YFP fluorescence was quantified using Image J software and expressed relative to untreated basal levels (set at 1).

When 1, 3 and 40 uM Dex was applied to pPopY-hpYFP roots, four days after induction fluorescence was reduced ~35%, 70% and 90% respectively relative to the absence of inducer (Fig. 3a), showing a dosage dependence on RNAi silencing. 100 uM Dex induced similar fluorescence reductions as 40 uM at each time point, indicating a saturation concentration of inducer (Fig. 3a). The application of 1 uM estradiol to pER8Y-hpYFP transgenics reduced YFP fluorescence about 35% while 10 uM estradiol reduced fluorescence about 45% relative to basal levels (Fig. 3c).

### Dexamethasone and estradiol induction rapidly reduces YFP mRNA levels

**Figure 4:**
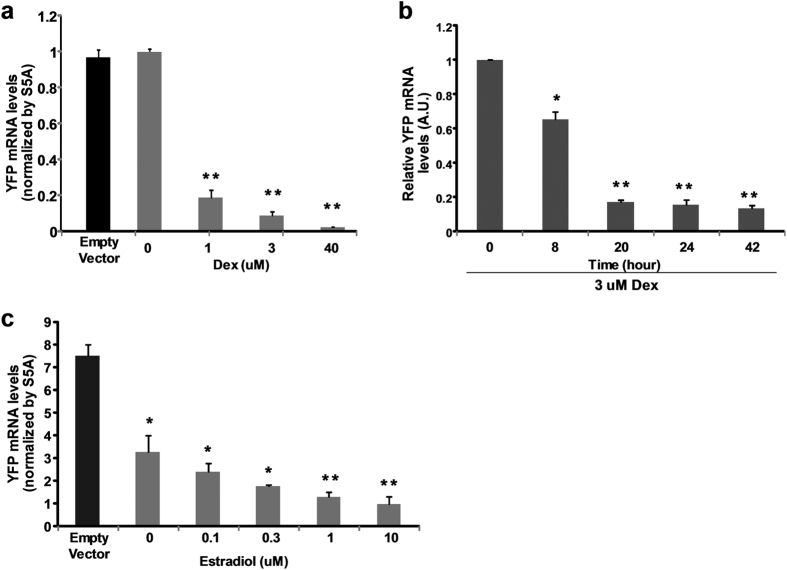
YFP mRNA levels after chemical induction. Transgenic Medicago roots were treated with different concentrations of inducers for various times before RNA was isolated and YFP transcript levels measured by RT-PCR. Proteasomal subunit S5A was included in the RT-PCR for normalization. Data expressed as mean +/− sem, (**a**) YFP mRNA levels after 6 days treatments by different concentrations of Dex on pPopY-hpYFP roots. n = 6. **p < 0.01, 0 vs. indicated Dex concentrations by t-test. (**b**) YFP mRNA levels at 0, 8, 20, 24 and 42 hours under treatment of 3 uM Dex. YFP mRNA levels were expressed relative to basal levels from hr 0, averaged and plotted. n = 6. *p < 0.05, **p < 0.01, time 0 vs. indicated time points. (**c**) pER8Y-hpYFP transformants were treated with 0, 0.1, 0.3, 1, 10 uM estradiol for 4 days before RNA was isolated and measured by RT-PCR. Data as mean +/− sem, n = 3. *p < 0.05, **p < 0.01, empty vector vs. estradiol treatments.

We evaluated the changes in YFP mRNA levels by quantitative RT-PCR after treatment with the chemical inducers. Six days after the treatments with increasing concentrations of Dex, YFP mRNA levels were reduced up to 97% comparing with non-induced basal levels (Fig. 4a). To further refine the kinetics of target mRNA reduction, we quantified the YFP mRNA levels at 0, 8, 20, 24, and 42 hours after 3 uM Dex treatment. Under these conditions, mRNA levels were significantly reduced 8 hours after induction and reduced to minimal levels by 20 hours (Fig. 4b). When comparing the reduction kinetics between YFP mRNA and YFP fluorescence at the measurement time points, at all Dex concentrations YFP mRNA reduced more rapidly than YFP fluorescence such as 3 uM Dex treatment (Fig. 3a and b; Fig. 4a and b).

As predicted from the fluorescence, there was a significant reduction in YFP mRNA in pER8Y-hpYFP roots even without chemical induction. However, the levels of YFP mRNA was further reduced in roots four days after the treatments with 1 and 10 uM estradiol (Fig. 4c).

As indicated in Fig. 4b, induction with 3 uM Dex reduced YFP mRNA about 50% in a little more than 8 hours while 50% reduction in fluorescence took about 3 days (Fig. 3a).

### Reversibility of silencing in absence of inducer

**Figure 5:**
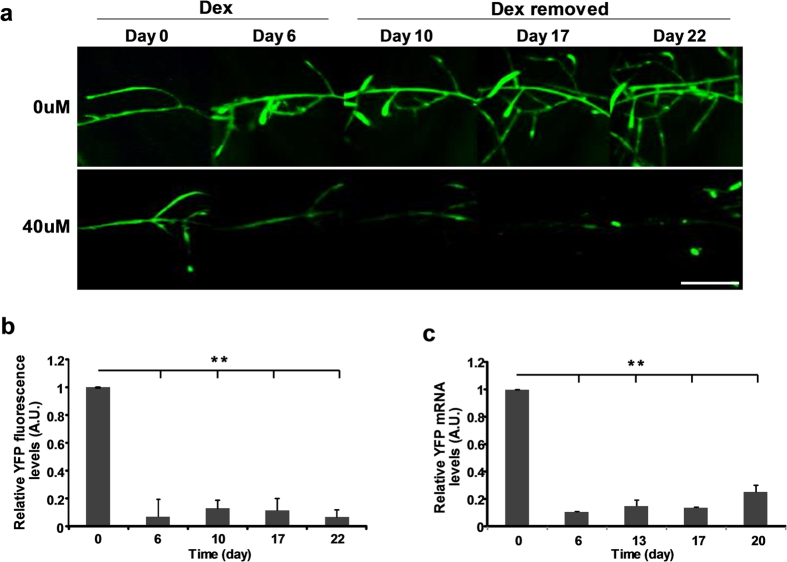
Reversibility of YFP in pPopY-hpYFP transformed roots after removal of Dex. (**a**) YFP images of transgenic roots before and after treatment with 40 uM Dex for 6 days. At day 7, roots were moved to media without Dex and YFP fluorescence monitored an additional 16 days. Scale bar = 10 mm. (**b**) Quantitation of YFP fluorescence before and after Dex removal. YFP fluorescence levels were quantified using Image J software, expressed relative to basal levels from day 0, averaged and plotted. Data expressed as mean +/− sem, n = 4. **p < 0.01, time 0 vs. indicated time points. (**c**) Quantification of YFP mRNA before and after Dex removal. Proteasomal subunit S5A was used for normalization. YFP mRNA levels were expressed relative to basal levels from day 0, averaged and plotted. Data expressed as mean +/− sem, n = 4. **p < 0.01, time 0 vs. indicated time points.

**Figure 6:**
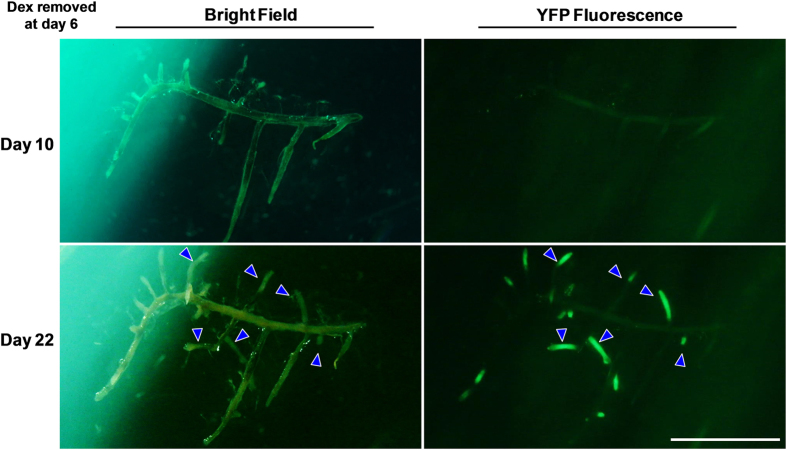
YFP is expressed in newly emerging roots. pPopY-hpYFP transgenic roots were photographed 2 and 16 days after removal from Dex. Four independent experiments were performed; representative images are shown. Left panel: bright field images. Right panel: YFP fluorescent images. Arrow heads: roots that develop after Dex removal. Scale Bar = 10 mm.

To examine the reversibility of silencing after induction of the pPopY-hpYFP vector, we moved the roots to new media without inducer after 6 days’ treatment. YFP fluorescence did not recover up to 16 days after removal of Dex (Fig. 5b), although non-treated YFP roots continued to show robust YFP expression (Fig. 5a). During the 16 days’ growth without Dex, we observed new lateral roots emerging; interestingly, these are YFP positive while old parental roots remain YFP silenced (Fig. 6).

To evaluate YFP mRNA levels after Dex is removed, we isolated mRNA from induced roots at different time points after inducer removal and performed RT-PCR. For at least 16 days after removal of Dex, YFP mRNA levels remained at similar levels as before Dex removal, except for the slight elevation in message levels at day 16 (Fig. 5c). It is likely that the newly emerged YFP positive roots accounts for the small increase in YFP mRNA at day 16.

### Chemical induction of a lethal gene in roots

We are characterizing these vectors in roots with the goal of generating transgenic plants with RNAi constructs targeting genes that are critical for root survival. As a proof of concept, we inserted a RNA hairpin structure targeting cytosolic acetyl-CoA carboxylase (AccASE) into pPopY-hpYFP and transformed it into Medicago roots. AccASE catalyzes the ATP-dependent formation of malonyl-CoA from acetyl-CoA and bicarbonate and is a key enzyme for de novo fatty acid synthesis^19^. We showed previously that certain construct silencing AccASE in Medicago inhibit root development^20^. We cloned a hp cassette targeting Medicago AccASE into the SfaAI site on the right side of the pOp6 promoter (Fig. 1a). This vector is called pPopY-hpACC. Medicago roots transformed by pPopY-hpYFP serve as controls.

**Figure 7:**
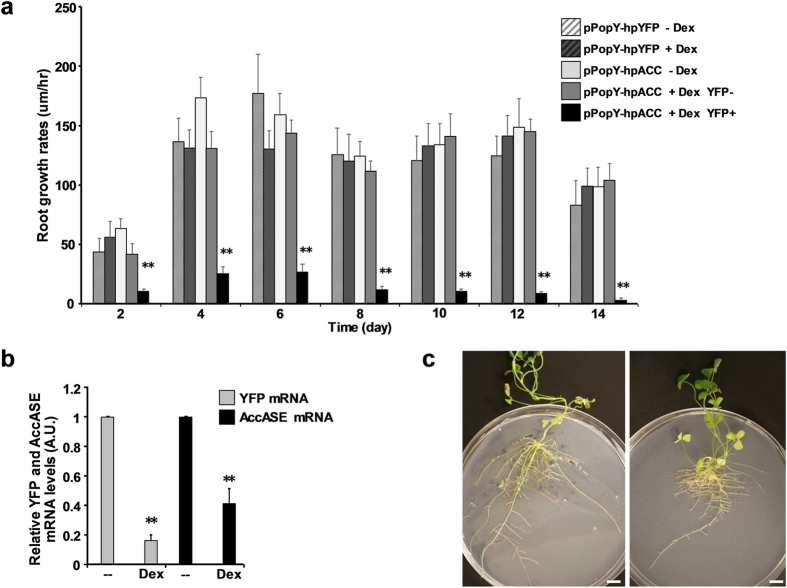
Chemically induced silencing of AccASE in Medicago roots. (**a**) pPopY-hpYFP or pPopY-hpACC roots were grown in Hoagland’s media with or without 50 uM Dex for 14 days. Roots were imaged and growth rates quantified by Image J software. Growth rates were expressed as um/hour, data shown is mean +/− sem, n = 20–40. YFP negative roots (YFP−) in pPopY-hpACC transformed plants are non-transgenic while “YFP+” YFP positive roots are transformed. **p < 0.01, pPopY-hpACC YFP+ vs. four control groups by t-test. (**b**) pPopY-hpACC transformants were grown in Hoagland’s media with or without 50 uM Dex for 24 hours. Total RNA was isolated and YFP and AccASE mRNA levels were determined by RT-PCR using proteasomal subunit S5A for normalization. YFP and AccASE mRNA levels were expressed relative to their own basal levels without Dex treatment, averaged and plotted. Data as mean +/− sem, n = 4. **p < 0.01, −Dex vs. +Dex by t-test. (**c**) Images of pPopY-hpYFP and pPopY-hpACC transformed plants grow in Hoagland media with 50 uM Dex. Scale Bar = 10 mm.

We examined root growth rates in Medicago transformed with pPopY-hpACC or pPopY-hpYFP before and after Dex induction. Transformed Medicago roots were grown in non inducing media for seven days; then pPopY-hpACC transgenic roots and controls, i.e. pPopY-hpYFP, were moved onto media with or without 50 uM Dex for continuing growth and photographing roots daily. Notably, although most pPopY-hpACC transgenic roots are YFP positive (marked as pPopY-hpACC YFP+) and they did grow out few YFP negative roots (marked as pPopY-hpACC YFP−), indicating the growth of the sporadic non-transformed root cells. After moving to the new plates, the initial growth of all the genotype plants slowed in presence or absence of Dex. From 4–6 days, all the plants increased the growth rates. Markedly, along the monitored growth at different time points, Dex treated pPopY-hpACC YFP+ roots consistently revealed greatly reduced the growth rates compared to the treated pPopY-hpACC YFP− roots, the untreated pPopY-hpACC roots and to the treated pPopY-hpYFP controls (Fig. 7a), suggesting that the AccASE hairpin in roots caused the growth reduction under Dex (Fig. 7a) (Fig. 7c image of whole plants). These results suggested that the root growth inhibitory gene hairpins in pPopY-hpACC can be transformed into roots in the absence of Dex and that the transformed roots can develop normally and become activated with inducer.

To validate the silencing of AccASE gene, we examined transcript levels of YFP and AccASE after the treatment of 50 uM Dex for 24 hours. Comparing with non-treated roots, Dex induced ~60% reduction in AccASE mRNA and ~85% reduction in YFP mRNA in pPopY-hpACC roots after 24 hours (Fig. 7b), suggesting that RNA hairpin cassettes on both sides of pOp6 promoter were induced, leading to the silencing of YFP and AccASE genes.

## Discussion

Zuo and Chua (2000) summarized important traits for a chemical inducible system^21^. These include low basal expression, high specificity, low toxicity, fast response, high dynamic range and reversion following removal of inducer. We characterized the modified Dex inducible pOpOff2 and estradiol inducible pER8 by these criteria.

In the absence of Dex, Medicago roots transgenic for pPopY-hpYFP had similar levels of YFP fluorescence and mRNA as transgenic roots without the hairpin. In contrast, pER8Y-hpYFP transformed roots had reduced fluorescence and reduced basal mRNA levels compared to empty vector controls. Other researchers have reported on the leakiness of pER8 in the absence of inducer^22^,^23^,^24^. Because animal steroids do not typically exist in plants, activation of steroid receptors should require exposure to endogenous steroids. However, legumes do contain significant concentrations of phytoestrogens which may be responsible for the expression from the pER8 promoter in Medicago. As observed in the above ground parts of Arabidopsis and tobacco, neither dexamethasone nor estradiol appeared to have effect on the growth of transgenic Medicago roots^3^,^10^,^11^.

Ideally chemical inducible promoters are sensitive to low concentrations of inducer and dosage dependent across a wide dynamic range. Previous studies showed GUS expression driven from pOp6 inducible promoter reached maximal levels 10 hrs after Dex treatment^10^,^11^. In our experiments the pOp6 inducible promoter drives transcription of an hpRNA that silences a target gene’s transcript levels. Under these conditions, expression of the target YFP transcript was reduced to its minimal levels 20 to 24 hour after induction. Further, similarly fast response and effective silencing were also observed when targeting a root growth gene, i.e. AccASE, in our modified vector.

In transgenic roots the pPopY-hpYFP vector was induced by Dex at concentrations ranging from 1 uM to 100 uM in a dose-dependent manner; no additional silencing was observed at higher concentrations (Fig. 3a). Our pPopY-hpYFP vector had overall a more gradual response to Dex concentrations in comparison with the other Dex reports^10^,^11^.

A previous report of the Dex inducible pOp6 promoter showed reversal of the silenced PDS gene mRNA, but not silenced LUC transgene mRNA, within 10 days after Dex removal^13^. In our experiments silencing of the marker transgene YFP was not reversed of the 22 days after Dex removal. Our observation is similar to the study of Wielopolska *et al*., 2005, i.e. unlike endogenous PDS gene, the silencing of LUC transgene was not reversed after removing Dex. Although the authors did not give reasons^13^, one other report did shed light on transgene silencing^25^. In that study, after infected with a RNA virus modified to carry part of GFP coding region, GFP was transcriptionally suppressed during and even after removing that RNA virus^25^. Continued silencing of GFP was reasoned to be caused by RNA-mediated DNA methylation (RdDM) at GFP coding region, thereby leading to sustained epigenetic inhibition of GFP transcription^26^. We postulate that Dex-induced siRNA against YFP mRNA triggered epigenetic modulation of YFP coding DNA, leading to sustained transcriptional repression even after Dex removal. We consider the alternative hypothesis that Dex is retained in the cells for prolonged time; this is unlikely because in Dex inducible pOp6-GUS ectopic expressing transgenic Arabidopsis, when Dex inducer was removed, the induced GUS transcript dropped within 24 hours, suggesting that Dex was not retained in the plants likely due to its degradation by metabolism or compartmentalization in plant cells^13^. Exact mechanism is warranted further investigations for YFP transgene.

The vector has a bidirectional pOp6 promoter that can drive transcriptions of two genes at once^10^,^11^. In our study, YFP and AccASE specific-RNA hairpins were cloned on either side of the pOp6 promoter (Fig. 1a and Fig. 7b), driving transcription of RNAi for both YFP and AccASE. In transformed roots, mRNA reductions in YFP and AccASE were not necessarily to the same extent. Similar poor correlation between the expressions of two transgene, i.e. GUS and Luciferase, engineered on both sides of pOp6 promoter was also observed^10^,^11^ and the authors proposed that other factors acting outside the operator array can additionally influence the expression of individual genes. Accordingly, in our modified inducible system, the silencing of two genes is determined by the transcription of each gene’s specific RNAi driven from either side of pOp6 promoter as well as subsequent RNAi mediated degradation of that gene’s transcript. Although the generations of RNAi from either side of pOp6 promoter can be independent, efficient silencing of YFP marker by our modified vectors provides a visual indicator for the activation of pOp6 bi-directional promoter. More importantly, the AccASE silencing reported here illustrates that the chemical inducibility can be used to conditionally silence essential genes after transformation.

When roots were exposed to 3 uM or more Dex, YFP mRNA was depleted within 24 hours while no significant changes of YFP fluorescence was observed during the same period of time. These data suggested that the YFP protein half-life in Medicago roots is around 2.5 days. Using a similar approach, the half-life of GFP in C. elegans was estimated around 1.5 days^27^ while bacterial GFP has a half-life of 9.5 days^28^. Clearly the GFP has varied half-lives depend on cell types and our Dex inducible silencing system allows an estimate of YFP fluorescent protein’s half-life in Medicago roots.

Although the application of dexamethasone system has been reported in leaves^3^, flowers^3^ and seeds^10^, less has been described on application of the inducible silencing systems in roots. Our data provided original quantitative analyses on how this chemically inducible system can silence genes in a temporal and dose-dependent manner in roots and demonstrated how a lethal gene could be chemically silenced and the resultant phenotypes illustrated in roots.

## Methods & Materials

### Plant Material and Rhizobial Strain

Seeds of the *Medicago truncatula* accession Jemalong A17 was obtained from Dr. Doug Cook (University of California, Davis). Agrobacterium rhizogenes strain MSU440 was used for root transformation.

### Modification of pOpOff2 and pER8 vectors for Transformation and RNAi

The vectors pOpOff2^13^ and pER8^4^ were obtained from Andrew Waterhouse (CSIRO) and Nam-Hai Chua (Rockefeller University) respectively. The gene encoding the yellow fluorescent protein (YFP) under the regulation of the mannopine synthase (MAS) promoter and nos 3′ terminator was PCR amplified from pHG8-YFP^20^ and cloned into the *AflII* site of pOpOff2 and the *PvuII* site of pER8. A hairpin cassette consisting of 373 bp of YFP sequences (base pairs 339–711) cloned as an inverted repeat flanking a CSHA intron was then cloned behind the inducible promoter in each plasmid. The GUS gene in the original pOpOff2 vector was deleted and replaced with a unique SfaA1 restriction enzyme site. The modified vectors were renamed pPopY-hpYFP and pER8Y-hpYFP respectively (Fig. 1a,b).

### *Medicago* root transformation

Root transformations were performed as previously described using the A. rhizogenes strain MSU440^20^. After inoculation with MSU440, chimeric plants with transgenic roots were incubated in a 25 °C growth room with a 16-h light period at 150E light intensity. Approximately 2 weeks after transformation, transgenic roots were identified by visualization of YFP fluorescence with a Zeiss Stemi SV11 dissecting microscope equipped with a YFP filter set with excitation HQ500/20. Seedlings with transgenic roots were transferred onto fresh plates containing sucrose at 7.5 g/liter and the antibiotic timentin (SmithKline Beecham Pharmaceuticals, Philadelphia) at 300 mg/liter. Six weeks after transformation, positive transformed roots were selected for experiments.

### YFP fluorescence quantitation assay

Transgenic roots were photographed under the dissecting fluorescent microscope with a Canon EOS 70D camera (Canon, Japan) and the intensity values (arbitrary units) of YFP fluorescence quantified by Image J software (NIH, Bethesda, MD; http://rsb.info.nih.gov/ij).

YFP intensity was determined by subtracting the background Region of Interest (ROI) adjacent to the YFP root ROI. Similar ROI calculations were done at different time points to quantitate YFP changes over time. The temporal changes in YFP fluorescent intensities were expressed relative to the basal YFP intensity prior to treatment.

Transformed roots from pPopY-hpYFP or pER8Y-hpYFP system were induced by indicated concentrations of Dex or estradiol respectively and photographed for YFP fluorescence daily. Reversibility of induction was investigated by transferring pPopY-hpYFP roots onto media without Dex or with 50 uM Dex and photographed an additional 16 days.

### Transcriptional analyses

For quantitative real-time PCR (RT-PCR) assay, total RNA was isolated from *Medicago* roots using the TRIzol reagent (Invitrogen, CA, USA), treated with DNase1, and further purified using RNeasy Mini Spin columns (Qiagen, Hilden, Germany). RNA (1ug) from each sample was converted to cDNA using High Capacity cDNA Reverse Transcription kit (Applied Biosystems, MA, USA). 4 ng of reverse-transcription reactions was used for each RTPCR with primers (shown in Supplementary Table S1) using an ABI 7300 quantitative PCR system (Applied Biosystems, Foster City, CA, U.S.A.). The cycle conditions were 50 °C for 2 min, 95 °C for 10 min, 40 cycles of 95 °C for 15 sec and 60 °C for 1 min. Melting curves of PCR products were obtained, and only those producing a single melting peak were considered. Target gene expression was normalized to the 26S proteasome regulatory subunit S5A (*Medicago* Gene Index TC108192) and a ubiquitin carrier protein (*Medicago* Gene Index TC176441) as reference genes^29^,^30^. Data were analyzed using deltaCt (cycle threshold) the comparative cycle threshold (Ct) method, ratio real-time PCR method by subtracting Ct of target gene from normalizing control, also known as the 2^−ΔΔCt^ method, was employed. Similar results were obtained for both reference genes (Supplementary Fig. S1), so the relative expression data presented here was calculated using TC108192.

### The root length measurements

Root growth was monitored by marking the plate at the position of the root tips and then measuring growth every 24 hours using Image J (NIH). For each genotype, 20–40 independent root replicas were measured. The root growth rate was defined by root length growth divided by the time.

### Statistical analysis

All the experiments were arranged in a complete randomized design with indicated independent replicates. Data are expressed as means +/− SEM for the roots from minimal three independent plants or as indicated. The data obtained were compared between either two different experimental conditions or two genotypes using a two-tailed Student’s *t*-test. Differences were considered significant at P < 0.05 and highly significant at P < 0.01.

## Additional Information

**How to cite this article**: Liu, S. and Yoder, J. I. Chemical induction of hairpin RNAi molecules to silence vital genes in plant roots. *Sci. Rep.* **6**, 37711; doi: 10.1038/srep37711 (2016).

**Publisher's note:** Springer Nature remains neutral with regard to jurisdictional claims in published maps and institutional affiliations.

## Supplementary Material

Supplementary Information
