# Supplementary material for: Chemical induction of hairpin RNAi molecules to silence vital genes in plant roots
Source: Sci Rep. 2016 Nov 29;6:37711. doi: 10.1038/srep37711 (PMC5127191; doi:10.1038/srep37711)
Supplement: Supplementary Information [file srep37711-s1.pdf]

Supplementary information for:

Chemical induction of hairpin RNAi molecules to silence vital genes in plant roots

Siming Liu and John I. Yoder*

Plant Sciences Department, University of California, Davis, CA 95616

* To whom correspondence should be addressed: Tel.: 530-752-1741;
Email: jiyoder@ucdavis.edu

Content:

Supplementary Figure S1
Supplementary Table S1

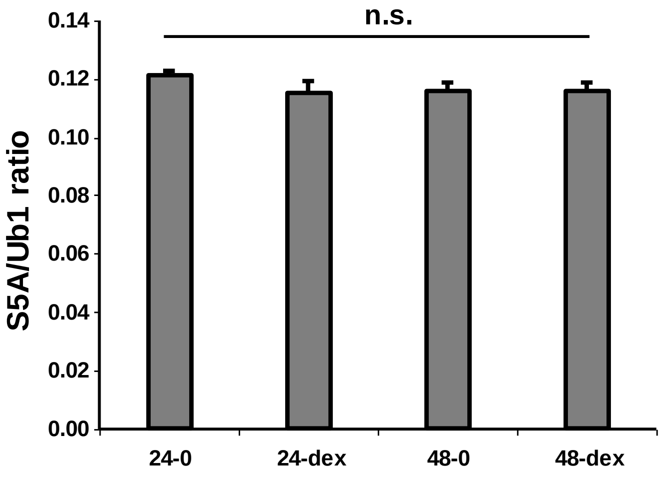

Supplementary Figure S1

Supplementary Figure S1. Ratios between the expressions of two reference genes do not change in Medicago roots treated with and without 50uM Dex.
To validate the stability of reference gene proteasomal subunit S5A under experimental conditions, total RNA was isolated from the transformed Medicago roots treated with 50 uM Dex or without Dex for 24 and 48 hrs. Expressions of Proteasomal subunit S5A (TC176441) and another reference gene, i.e. Ubiquitin carrier (TC108192), were examined by RT-PCR. The ratios of two genes' Ct values were plotted and were shown to be consistent under all the indicated treatment conditions, n=3; n.s., no significance.

Supplementary Table S1. Primers used in RT-PCR.

Primer name	Forward primer (5'-sequence-3')	Reverse primer (5'-sequence-3')
YFP	GTGGTGCCCATCCTGGTCGA	AAGTCGTGCTGCTTCATGTGG
Medicago AccASE	GAGGGTTGTTCCTCAAGCTG	TTCGTTCGGCATACATTTCA
26S Proteasome regulatory subunit S5A	TGGCAGGAAAGGGTGTTC	GCCACCTGAATACCAGCAG
Ubiquitin carrier	GATCCAAATCCCGATGAC	CGGTGGCTTCATACTTGGTC
